# Anxiety and depression among various blood groups of undergraduate medical and physical therapy students

**DOI:** 10.12669/pjms.40.10.8984

**Published:** 2024-11

**Authors:** Rubab-e-Hira, Muhammad Ali, Shireen Jawed, Benash Altaf

**Affiliations:** 1 Rubab-e-Hira, Final Year MBBS Aziz Fatimah Medical and Dental College, Faisalabad, Pakistan; 2 Muhammad Ali, Final Year MBBS Aziz Fatimah Medical and Dental College, Faisalabad, Pakistan; 3 Shireen Jawed, Associate Professor, Department of Physiology. Aziz Fatimah Medical and Dental College, Faisalabad, Pakistan; 4 Benash Altaf Assistant Professor, Department of Physiology, Aziz Fatimah Medical and Dental College, Faisalabad, Pakistan

**Keywords:** Depression, Anxiety, Blood Group, Medical students

## Abstract

**Objective:**

To access the frequency of depression and anxiety among the various blood groups of medical and physiotherapy students.

**Method:**

A cross-sectional study on 215 MBBS and DPT students was conducted at Aziz Fatimah Medical and Dental College (AFMDC) from November 2022-May 2023. Google Form containing sections for relevant information concerning participants and fourteen items- Hospital anxiety and depression Scale (HADS) questionnaire for evaluation of anxiety and depression, was administered among the MBBS and DPT students through social media. The data was analyzed using SPSS version 26. Mean ±SD was calculated for continuous variables like HADS score and age. Frequency and percentage were calculated for categorical variables such as blood groups, anxiety and depression. ANOVA was used to compare HADS score among various blood groups. P-value ≤0.05 was taken statistically significant.

**Result:**

Mild anxiety was found in A>O>B>AB, while severe anxiety was found in B>A>AB>O. Mild depression was found in A>B>O>AB, while severe depression was found in A>O>B>AB. However, on ANOVA, when comparing anxiety and depression scores with blood groups, they were found to be higher in blood Group-A. Rhesus factor positive blood groups have severe anxiety and mild depression while Rhesus factor negative blood groups have mild anxiety and severe depression.

**Conclusion:**

Severe anxiety was common in blood Group-B while severe depression was common in people with blood Group-A. While mild anxiety and mild depression was found in blood Group-A, however, sever anxiety was common in positive Rhesus factor blood types while severe depression was found in negative Rhesus factor blood types.

## INTRODUCTION

Stress Anxiety and depression are the most common health concerns among medical students. Stress is the body’s response to different situations.^1^ It is a sign that requires attention. Medical education is a very stressful academic curriculum. It has many negative effects on the student’s mental health. Stress developments have far reaching implications both personally and professionally, attributed to factors including family and personal history of depression, economic instability, diagnosis of a serious illness, death of a loved one, separation of parents leading to alcohol consumption and suicidal attempts among medical students.^2^ Fear of professional exams, expectations of parents, and a lack of time for activities other than studies are few out of many factors that contribute to the development of stress.^1^

It can be categorized as good stress and the bad stress. Good stress motivates a person to do his best in attaining best outcomes and if the person is unable to handle good stress in order to attain his desired goals, then it is evolved into anxiety and depression. Depression is a primary cause contributing to disability globally and significantly adds to the entire burden of disease. The consequences of depression can drastically hamper a person’s cognitive abilities to experiences a fulfilling and rewarding life.

Many studies have been found to show the association of blood group type with the spread of illnesses like tumor burden, GERD, hematological disorder including coagulation, bleeding and clotting disorders, infectious and renal diseases.^3^ However, there are very few studies related to blood Group-Associations with stress, depression, and anxiety in Pakistan. The frequency of stress, anxiety, and depression among medical students in Pakistan ranges up to 74%.^4^ This study was carried out to check the level of stress in medical and physiotherapy students with different blood groups. The objective of our study was to explore frequency of depression and anxiety among the various blood groups of medical and physiotherapy students.

## METHODS

This cross-sectional study was conducted at Aziz Fatimah Medical and Dental College (AFMDC), Faisalabad, Pakistan.

### Ethical approval:

The study was approved by Institutional Ethics Committee of AFMDC (IEC/179-22, dated August 15, 2022) from November 2022-May 2023.

The sample size was calculated with prevalence 15% anxiety and depression among medical students, 95% confidence level and margin of error 5% using open epi sample size calculator.^4^ Estimated sample size was 196 participants; however, it was increased to 215 participants in order to gather maximum data to enhance validity and generalizability.^4^ Students of MBBS and DPT of both genders from first year to final year were included. Students already diagnosed with anxiety, depression, psychological and, behavioral disorders, or taking medicines for such cases were excluded from the study. Sample of 215 MBBS and DPT students were enrolled by convenience sampling technique. Validated and reliable questionnaire called the Hospital anxiety and depression Scale (HADS) questionnaire was used to assess depression and anxiety through Google survey. HADS is a self-assessment fourteen items questionnaire with seven items each for depression and anxiety subscales. Each item is rated on a four-point Likert scale (range 0–3), giving maximum scores of 21 for anxiety and depression each. A total subscale score of >8 points out of 21 maximum score considered to be case of anxiety or depression.^5^ A Google survey form was circulated among the students using social media, informing them about the study and its objectives. They were then requested to fill the form if they agreed to participate in this research. They were informed that their participation was voluntary and their identities would be kept anonymous. Relevant information, like age, gender, discipline (MBBS or DPT) academic years and blood groups were asked from the participants through the administered Google Form The total time taken to fill out the questionnaire was approximately 10 minutes.

### Statistical analysis:

Statistical analysis was done using SPSS 26.0. Mean±SD was calculated for continuous variables like HADS score and age. Frequency and percentage were calculated for categorical variables such as blood groups, anxiety and depression. HADS scores were compared among various blood groups using ANOVA. Level of significance was set at p-value ≤0.05.

## RESULTS

This study was comprised of 215 participants with mean age 21.28±1.834 years. Of total study participants 167(77.7%) were from MBBS and 48(22.3%) were belonged to DPT. In Current study 109 (50.7%) were female, whereas 106(49.3%) were male students. Most common ABO blood group noticed was blood Group-B followed by blood groups O and A, however least common blood group was AB among the study participants. Frequencies and percentages of A, B, AB and O blood groups were 53(25%), 84(39%), 24(11%), 54(25%) respectively. Concerning rhesus (Rh) blood group, 180(83.7%) students were Rh positive and 35(16.3%) were Rh negative.

We found that in the studied population, mild anxiety was found frequently in Blood Group-A (30.2%), followed by Blood group O (29.6%). However, severe anxiety was found in Blood Group-B (42%), followed by blood Group-A (37%), (Table-I(a)). Our results are validated by observing scoring, showing that Blood Group-A shows overall higher anxiety scores, followed by Blood Group-B’ and ’O’, and these anxiety scores are found to be statistically significant P=0.009, when mean scores are compared with ANOVA (Table-I(b)). Post hoc analysis also showed significant result for anxiety score shows P-value= 0.05. Blood-A shows more overall anxiety score, followed by B and O. Post hoc test for multiple comparison of mean shows significant difference among blood group ’B’ and ’O’ (P-value = 0.05).

**Table-I(a):** Frequency and percentages of anxiety in the ABO blood group.

ABO blood groups (n=215)	Anxiety	Frequency N (%)	Percentages (%)
A blood group (n=53)	Normal	17(32.1%)	32.1
	Mild anxiety	16(30.2%)	30.2
	Severe anxiety	20(37.7%)	37.7
B blood group (n=84)	Normal	30(35.7%)	35.7
	Mild anxiety	18(21.4%)	21.4
	Severe anxiety	36(42.9%)	42.9
AB blood group (n=24)	Normal	12(50.0%)	50.0
	Mild anxiety	5(20.8%)	20.8
	Severe anxiety	7(29.2%)	29.2
O blood group (n=54)	Normal	23(42.6%)	42.6
	Mild anxiety	16(29.6%)	29.6
	Severe anxiety	15(27.8%)	27.8

**Table-I(b):** Comparison of Anxiety Scores by ANOVA (n=215).

ABO blood groups	Frequency N (%)	Anxiety Scores Mean ±SD	P-value
A blood groups (n=53)	53(24.7%)	9.36±3.633	0.009
B blood groups (n=84)	84(39.1%)	9.32±4.105	
AB blood groups (n=24)	24(11.2%)	7.13±4.276	
O blood groups (n=54)	54(25.1%)	8.43±3.755	

Mild depression had found most commonly in students who have Blood Group-A (35%), followed by Blood Group-B, O and AB respectively, while severe depression was also commonly found in blood Group-A (30%), followed by Blood Group-O (24.1%), B and AB (Table-II(a)). Overall mean depression score was found higher in blood Group-A followed by blood group ’B’ but these results are not statistically significant (P=0.25), (Table-II(b)).

**Table-II(a):** Frequency and percentages of depression in the ABO blood group.

ABO blood groups (n= 215)	Depression	Frequency N (%)	Percentages (%)
A (n=53)	Normal	18(34.0%)	34.0
	Mild depression	19(35.8%)	35.8
	Severe depression	16(30.2%)	30.2
B (n=84)	Normal	39(46.4%)	46.4
	Mild depression	25(29.8%)	29.8
	Severe depression	20(23.8%)	23.8
AB (n=24)	Normal	14(58.3%)	58.3
	Mild depression	5(20.8%)	20.8
	Severe depression	5(20.8%)	20.8
O (n=54)	Normal	29(53.7%)	53.7
	Mild depression	12(22.2%)	22.2
	Severe depression	13(24.1%)	24.1

**Table-II(b):** Comparison of Depression Scores by ANOVA.(n=215)

ABO blood groups	Frequency N (%)	Depression Scores Mean ±SD	P-value
A blood groups (n=53)	53(24.7%)	8.42±4.439	0.250
B blood groups (n=84)	84(39.1%)	7.85±3.801	
AB blood groups (n=24)	24(11.2%)	7.08±4.042	
O blood groups (n=54)	54(25.1%)	7.76±4.166	

As regards Rhesus blood types, it was seen that subjects who were Rhesus positive blood type had severe anxiety whereas mild anxiety was found more in negative Rhesus blood group (Table-III(a)). These results are not found to be statistically significant concerning scores (P= 0.760), (Table- III(b)). Severe depression is most commonly found in negative Rhesus Blood group while mild depression were found in positive Rhesus blood group (Table- IV(a)). This finding is authenticated by depression scores as we found higher depression scores in negative Rhesus blood groups. But these findings are not statistically significant (P= 0.87), (Table- IV(b)).

**Table-III(a):** Frequency and percentages of anxiety in the RH factor.

Rh factor (n=215)	Anxiety	Frequency N (%)	Percentages (%)
Rh+ (n=180)	Normal	72(40.0%)	40.0
	Mild anxiety	41(22.8%)	22.8
	Severe anxiety	67(37.2%)	37.2
Rh- (n=35)	Normal	10(28.6%)	28.6
	Mild anxiety	14(40.0%)	40.0
	Severe anxiety	11(31.4%)	31.4

**Table-III(b):** Comparison of Anxiety Scores by ANOVA of Rh factor.

Rh factor	Frequency N (%)	Anxiety Scores Mean ±SD	P-value
Rh+	180(83.7%)	8.66±4.016	0.760
Rh-	35(16.3%)	8.54±4.252	

**Table-IV(a):** Frequency and percentages of depression in the Rh factor.

Rh factor (n= 215)	Depression	Frequency N (%)	Percentages (%)
Rh+ (n=180)	Normal	87(48.3%)	48.3
	Mild depression	52(28.9%)	28.9
	Severe depression	41(22.8%)	22.8
Rh- (n=35)	Normal	13(37.1%)	37.1
	Mild depression	9(25.7%)	25.7
	Severe depression	13(37.1%)	37.1

**Table-IV(b):** Comparison of Depression Scores by ANOVA of Rh factor.

Rh factor	Frequency N (%)	Depression Scores Mean ±SD	P-value
Rh+	180(83.7%)	7.67±4.094	0.877
Rh-	35(16.3%)	8.46±4.097	

## DISCUSSION

This study highlighted the importance of blood groups for developing anxiety and depression. Most common ABO blood group found among the current study participants was blood Group-B followed by Blood Groups-O and A, however least common blood group was AB among the study participants. Rh positive phenotype was more commonly distributed as compared to the Rh negative phenotype in current study subjects. Similar trends of distribution of ABO and Rh blood groups were reported by the previous studies conducted in Pakistan.^6^,^7^

The studied subjects with blood Group-A followed by Blood Group-O had mild anxiety and majority of them fall in severe depression, reflecting that these subject did not cope with anxiety, so they are prone to develop depression than other blood groups. The possible mechanism of this fact is exemplified by Sherrington et al., who suggested that Blood Group-O individuals did not cope to stress because they have more difficulty in catecholamine’s clearance once produced during stress. This study also explain that blood Group-A individuals respond to stress by releasing profuse adrenaline but elimination is also very fast. However, this explanation concerning blood Group-A does not matches with our results as we observed severe depression among A blood group.^8^

The current study shows that severe anxiety is common in blood Group-B. Our results are validated by anxiety scores, showing higher scores in blood Group-B. These results are not in line with the study conducted by Zadeh, S Z. M et al., which revealed no significant difference between blood groups in terms of anxiety.^9^

In contrast to listed findings, the study carried out by Pisk SV et al. indicated that stress and anxiety leading to psychiatric symptoms appear almost three times more frequently in people with the AB blood group than in people with other blood groups.^10^

Considering the depression, current study found that people with blood Group-A followed by blood group O suffer from severe depression more than people with other blood groups, who also suffer from depression but to a mild to moderate degree. Our results are validated by scores of depression; showing higher depression scores in blood Group-A.

These results are consistent with the study conducted by Cattell et al., indicating that the rate of depression in blood Group-A followed by blood group O is significantly higher than the AB and B blood groups.^11^ However, results of Zadeh, S Z. M et al. are not in agreement with the present study who did not find any significant relationship between types of blood Group-And depression.^9^ However, a study by Singg SLJ et al. Reported higher depression scores among US adult with type O blood group.^12^ In contrast to current study results, previous study conducted in Iran by Romian et al. did not find significant difference concerning anxiety and depression among various blood groups.^13^

Kudlow P observed that the elimination of nitrogen oxide is more in carriers of B and AB blood groups than in other blood group individuals; this neurotransmitter is considered to be responsible for the development of depression and other psychiatric symptoms because it is a crucial neuro-inflammatory regulator.^14^

Interesting results had been documented by Agrawal M et al. study conducted at India indicating higher rates of psychological morbidity like anxiety/depression among the young adults having blood Group-A.^15^ In contrast to our findings which depict severe depression and severe anxiety in negative and positive Rhesus respectively, results documented by Agrawal M et al, indicated that there were no significant differences concerning occurrence of stress and depression among Rh positive and negative individuals.^15^

Due to high frequency of psychological morbidity like anxiety/depression among the young students there is a need of prompt identification of high risk individuals for timely management along with other health problems.^16^ Moreover, appropriate educational interventions especially parent institute motivation and counseling as well as instructional design in medical curricula are needed to improve the attitudes of our potential doctors and strengthen the healthcare system.^17^-^18^ In addition, steps for encouraging students to timely seek help from psychologist should be promoted as majority of our society consider that visiting psychologist as a stigma. Hence, steps should be taken on broader scale to de-stigmatize seeking help of psychologist.^19^ Future studies are recommended on broader scale to validate our findings concerning anxiety and depression among various blood groups.

### Limitation:

It is a single center study with small sample size.

## CONCLUSION

Severe anxiety was common in blood Group-B while severe depression was common in people with blood Group-A, while mild anxiety and mild depression was found in blood Group-A However, sever anxiety was common in positive Rhesus factor blood types while severe depression was found in negative Rhesus factor blood types.

### Author’s Contribution:

**RH and MA:** Study concept, study design, data collection, manuscript writing and approval of manuscript.

**BA and SJ:** Study design, study concept, data analysis, interpretation of results, critically revised and approved the article.

All authors are accountable for accuracy and integrity of the data.
